# Edible Insects as an Alternative Source of Nutrients: Benefits, Risks, and the Future of Entomophagy in Europe—A Narrative Review

**DOI:** 10.3390/foods14020270

**Published:** 2025-01-15

**Authors:** Wojciech Michał Jankowski, Dominik Przychodniak, Weronika Gromek, Emilia Majsiak, Marcin Kurowski

**Affiliations:** 1 Department of Immunology and Allergy, Medical University of Lodz, 90-419 Lodz, Poland; wojciech.jankowski1@student.umed.lodz.pl (W.M.J.); dominik.przychodniak@student.umed.lodz.pl (D.P.); weronika.gromek@student.umed.lodz.pl (W.G.); 2 Student Scientific Association for Allergy, Asthma, and Immunology, Department of Immunology and Allergy, Medical University of Lodz, 90-419 Lodz, Poland; 3 Polish-Ukrainian Foundation of Medicine Development, Nałęczowska 14, 20-701 Lublin, Poland; 4 Department of Health Promotion, Faculty Health of Sciences, Medical University of Lublin, Staszica 4/6, 20-081 Lublin, Poland

**Keywords:** allergen cross-reactivity, edible insects, entomophagy, food allergy, molecular allergy diagnosis

## Abstract

According to projections by the Food and Agriculture Organization of the United Nations, the global population will reach 9 billion by 2050. This raises concerns about the ability to feed such a population. In view of the above, it is necessary to search for alternative food sources. Edible insects are rich in complete protein, essential fatty acids, vitamins and micronutrients. Despite this, entomophagy is not common in Europe. In 2021, the European Union approved *Acheta domesticus*, *Tenebrio molitor*, *Locusta migratoria*, and *Alphitobius diaperinus* for consumption. However, their consumption may also be associated with certain hazards, e.g., food allergies. The purpose of this review is to present existing knowledge, discuss the possible dangers of consuming insects, and identify areas for further research. Studies in Asian populations indicate that edible insects may be responsible for 4.2–19.4% of food allergies and 18% of fatal food-induced anaphylaxis. There are also increasing reports from Europe of food allergies to edible insects. A thorough understanding of allergens, their properties, and the mechanisms of food allergies associated with edible insects’ consumption is essential for ensuring consumers’ safety. In the future, it would be worthwhile to investigate the effects of heat treatment on the allergenicity of insect proteins.

## 1. Introduction

Food allergy is defined as a syndrome of adverse symptoms that develop because of an excessive immune response following the ingestion of a food containing a specific allergen or allergens [1]. Given the marked increase in the prevalence of allergic disease over the past two decades, it is now regarded as a significant public health issue [2]. It is estimated that 8% of children and 10% of adults are affected by this condition [3]. The rise in the number of new cases identified can be attributed to the hygiene hypothesis [4]. The hypothesis posits that a lack of exposure to microorganisms and parasites during early childhood can contribute to an increased prevalence of allergic and autoimmune diseases [4]. The symptoms of food allergy may include swelling (including of the throat), disrupted intestinal peristalsis, runny nose and wheezing, but most often manifest as cutaneous reactions in the form of pruritus and rash [4]. The most severe manifestation of an allergic reaction is anaphylactic shock [5]. Allergies typically emerge through one of two mechanisms: type I hypersensitivity or type IV hypersensitivity [4]. Type I hypersensitivity, also referred to as immediate hypersensitivity, is initiated by type E immunoglobulins (IgEs), which, when complexed with an allergen, stimulate the degranulation of mast cells and basophils, resulting in the release of inflammatory mediators, including histamine. These are responsible for the symptoms of an allergic reaction [4]. In contrast, type IV hypersensitivity, also referred to as delayed hypersensitivity, is regulated by allergen-activated T lymphocytes, which stimulate the production of inflammation approximately 24–48 h after exposure, leading to tissue damage [4]. The majority of allergies to edible insects are of the IgE-mediated type [6].

Currently, there is no cure for food allergies, and thus it is recommended that individuals with food allergies eliminate products containing the allergen from their diet [3,5].

**Figure 1:**
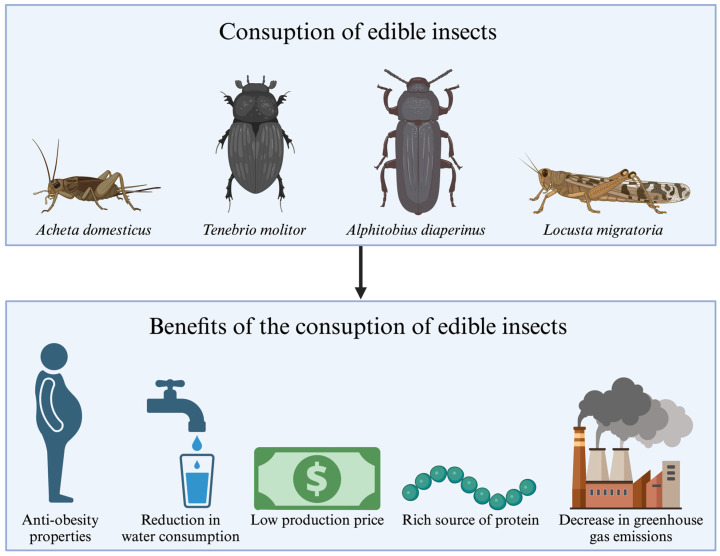
Potential benefits of including edible insects in one’s diet.

As projected by the Food and Agriculture Organization of the United Nations (FAO), the global population is expected to reach 9 billion by 2050, a figure that gives rise to concerns regarding the capacity to feed such a vast number of individuals [7]. In light of this, it appears imperative to pursue the utilization of alternative food sources that are distinguished by their economic and ecological sustainability (see Figure 1) [7]. Insect-based food production offers substantial environmental advantages, including a reduction in water consumption and a decrease in greenhouse gas emissions [8].

In developed countries, the problem has shifted from a shortage of food to the consumption of excessive amounts of unhealthy food, resulting in obesity and leading to the development of metabolic diseases. The results of animal studies suggest that the consumption of edible insects is characterized by anti-obesity properties, such as lipid-lowering and appetite-regulating effects. These properties allow for the potential use of edible insects in modern dietary interventions [9]. Moreover, edible insects are characterized by high nutrients and vitamins content, making them a valuable food source [8,10]. For these reasons, edible insects are seeing increasing utilization in the food industry, primarily as food additives to augment their nutritional value. One illustrative example is protein bars, which employ protein derived from crickets. Cricket flour is utilized in the production of pasta. Insects are also employed as a meat substitute for sauces. They are also utilized in the manufacture of snacks [11].

However, it should be noted that the consumption of edible insects may also present certain risks, including the potential for food allergies [7]. The objective of this review article is to synthesize the extant knowledge on the consumption of insects, to delineate the potential risks associated with this practice, and to suggest avenues for further research in this area

## 2. Edible Insects and Their Allergens—General Aspects

Edible insects are distinguished by their high protein content, which includes essential amino acids such as omega-3, as well as B vitamins and micronutrients [8,10]. Approximately two billion people consume insects, primarily residing in regions of Asia, South America, and Africa [1]. It is estimated that approximately 2000 distinct species of insects are consumed worldwide [1,12]. Nevertheless, entomophagy (the practice of consuming insects) remains relatively uncommon in Europe [1]. This is a relatively recent phenomenon, occurring only in 2021. The European Union has granted approval for the consumption of *Tenebrio molitor*, *Acheta domesticus*, *Locusta migratoria* and *Alphitobius diaperinus* [12]. They are primarily utilized as enrichment ingredients in food products [7]. Such products are incorporated into protein bars, snacks, pasta, and baked goods, among other items.

However, it is important to note that the consumption of insects is not without associated risks. These include chemical risks, such as the potential accumulation of heavy metals and anti-nutritional substances, microbiological risks, and allergic risks [7,13]. The current findings, based on Asian populations, indicate that edible insects are responsible for 4.2–19.4% of food allergies and 18% of cases of fatal anaphylaxis caused by a food allergen [14]. Furthermore, an increasing number of reports from European countries have documented food allergies to edible insects [1].

Allergic reactions following the ingestion of insects may result from cross-reactivity or primary sensitization [7]. The initial symptoms typically manifest within minutes to six hours [1].

The phylogenetic affinity between insects, crustaceans, and the house dust mite can result in cross-reactivity [14]. Such cross-reactivity may be exemplified by the situation in which patients who are allergic to shrimp are more susceptible to developing an allergic reaction to edible insects, and vice versa, i.e., that individuals allergic to edible insects may demonstrate an elevated proclivity for developing allergy after shrimp consumption. Common allergens, which are present in multiple species of the invertebrates and involved in the development of cross-reactivity, include tropomyosin, arginine kinase, and glyceraldehyde 3-phosphate dehydrogenase.

Primary sensitization can be induced by several allergens, including arginine kinase, tropomyosin, aspartate protease, hemocyanin, glutathione S-transferase, troponin C, myosin light chain, serine protease, and α-amylase [11].These allergens can be identified using laboratory tests such as Western blotting, which detects proteins opsonized by IgEs solubilized in the plasma of patients with food allergies [15].

In this review we present the main allergens of edible insects as well as their sources and physicochemical and allergenic properties. Subsequently, we address the issue of the potential of edible insects’ allergens to induce clinical allergy symptoms resulting from cross-reactivity with allergens originating from other sources. Further, the current diagnostic options are discussed and finally, existing knowledge gaps and research needs are highlighted.

## 3. Methodology

A search of the online databases PubMed, Scopus, and Google Scholar was conducted between May and August of 2024 using the search terms: “edible insects”, “entomophagy”, “food allergy”, “*Acheta domesticus*”, “*Locusta migratoria*”, “*Tenebrio molitor*” and “*Alphitobius diaperinus*”. The logical operators “and/or” were used to narrow the search results. The primary objective was to identify articles describing food allergies to edible insects available in the European Union as well as in other countries, identifying possible geographically determined differences in edible insects’ spread and application in the food industry. Secondary search objectives included identifying articles describing the allergenic potential of edible insects, with a focus on their potential to elicit cross-reactions between edible insects’ allergens and allergens coming from other, mostly unrelated species. Subsequently, WMJ and DP conducted an independent, two-fold subjective evaluation of the identified publications. Initially, the titles and abstracts were assessed, and subsequently, the full texts were reviewed. Considering these considerations, the articles included in the review were selected. Only articles published in peer-reviewed journals in English were considered for inclusion in the review.

## 4. Edible Insects and Their Allergens—Detailed Characteristics

Currently, the European Commission authorizes the presence of four species of edible insects on the food market of the European Union. These are *Acheta domesticus* [16], *Tenebrio molitor* [17], *Locusta migratoria* [18] and *Alphitobius diaperinus* [19].

### 4.1. Acheta Domesticus (The House Cricket)

The incidence of food allergies caused by the consumption of *Acheta domesticus* is on the rise, largely due to the growing interest in insects as a potential source of protein [20]. Dried crickets contain approximately 60.26 g of protein per 100 g of product, which is a higher protein content than that of dried chicken breast, which contains approximately 50 g of protein per 100 g of product. The fat content of dried crickets is typically in the range of 10.18 g per 100 g of product. Additionally, they are a rich source of B vitamins, iron, zinc, and other micronutrients [21].

It is of paramount importance to identify the primary allergens present in crickets and to ascertain the impact of diverse heat treatments on these allergens. This knowledge is vital for the safe integration of these insects into the human diet. The application of heat treatments has the potential to diminish the allergenicity of *Acheta domesticus* proteins. This can be achieved by denaturing certain proteins, such as arginine kinase, while others, such as tropomyosin, remain extremely resistant to high temperatures (Table 1) [20].

### 4.2. Tenebrio Molitor (The Yellow Mealworm)

The mealworm is a rich source of complete proteins, essential fatty acids, fiber, and micronutrients. Dried whitefly larvae contain 49.1 g of protein, 38.4 g of fat, and 8.5 g of carbohydrates per 100 g of product, as reported in reference [14]. It is of paramount importance to identify the primary allergens present in whitefly species and to assess the impact of thermal processing techniques on their allergenic potential. This is a crucial step towards the safe incorporation of these insects into the human diet. Some mealworm allergens become denatured and less allergenic due to thermal processing, while others remain unaffected even by very high temperatures and pressure, maintaining their stability (Table 2) [23].

### 4.3. Locusta Migratoria (The Migratory Locust)

Locust is a rich source of protein and fat, with a concentration of 70 g and 14 g per 100 g of dried product, respectively [26]. The calorific value of the product is approximately 400 kcal per 100 g of dry matter [26]. Additionally, the locust contains substantial quantities of trace minerals (such as calcium, iron, and zinc), vitamins (including D3, B12, E, and A), and essential fatty acids (Omega-3 and Omega-6) [26]. The consumption of locust has been demonstrated to exert a protective effect on the cardiovascular system due to the presence of phytosterols and proteins that exhibit angiotensin-converting enzyme inhibitory activity [26]. It is of the utmost importance to identify and understand the allergens present in *Locusta migratoria* to guarantee the safe incorporation of these insects into the human diet (Table 3).

### 4.4. Alphitobius Diaperinus (The Lesser Mealworm)

Lesser mealworms are considered to be a high-quality food source due to their high nutritional value resulting from their high protein content, as well as other components (fat, fiber, vitamins and minerals) [29]. Protein levels in lesser mealworms range between 45% and 60% depending on their stage of development and rearing conditions. Dried lesser mealworms contain 58–65% crude protein, an amount comparable to that identified in other edible insects that have been approved for human consumption [30]. The most abundant proteins in the lesser mealworm are actin, myosin and tropomyosin [31]. Another feature making lesser mealworms a valuable and high-quality food source is the fact that they contain all necessary amino acids in desirable and adequate amounts (as reviewed by Siddiqui et al. [29]). The allergens present in *Alphitobius diaperinus* are listed in Table 4; however, it is important to note that this species is the least studied among the insects analyzed so far.

## 5. Edible Insects Outside Europe

Consumption of edible insects is far more popular and rooted in culture and alimentary habits outside Europe, in Asia, Africa and Latin America, while the degree of acceptability of edible insects as food products in Europe is still relatively low and negative affective reactions towards them are widely encountered [32,33,34,35,36]. Examples of edible insects that are widely consumed outside Europe include beetles, caterpillars, ants, bees, wasps, grasshoppers, true bugs, dragonflies, termites, and cockroaches [8,37,38,39]. In contrast with Western countries, in those regions, edible insects are commonly consumed not only as sources of protein added to more “traditional” alimentary products, but also as traditional snacks and street food. Usually, they are cleaned, cooked and seasoned, before being processed into snacks through frying, grilling, fumigating or deep-frying—processes that add to their appearance, crispness and tastefulness, thus making them more attractive to consumers (as stated by Li et al. [38]). In this context, one should not forget the fact that thermal processing of edible insects may modify the allergenicity of edible insects’ proteins, contributing to modified risk of food hypersensitivity reactions and to modification of potential cross-reactive properties [1,40,41].

As is the case with other foods, consumption of edible insects and worms is influenced not only by culture, tradition and climate, but also by religious rules. This can be exemplified by different attitudes to considering edible insects themselves and insect-derived products as halal by different Muslim schools. The locust is an edible insect, the consumption of which is unanimously approved and supported among all Muslim scholars (reviewed by Suresh et al. [42]).

## 6. Epidemiology of Allergy to Edible Insects

Given the recent introduction of the possibility of using certain edible insects for food in the European Union, data on the epidemiology of food allergies in this context remain insufficient and require further research. Most available data are based on studies conducted in Asia.

In Asia, it is estimated that between 7.6% and 22.2% of people who eat edible insects have experienced the occurrence of symptoms related to allergic reactions caused by eating these products [14]. The most common allergic reactions following the ingestion of edible insects in the Asian population include urticaria, edema, dyspnea and anaphylaxis [14]. Similarly, anaphylaxis caused by eating edible insects accounts for up to 19.4% of all recorded cases of food anaphylaxis [43].

One of the challenges in applying the findings of this research to European contexts is the existence of a significantly broader range of edible insects in Asia, along with a greater prevalence of entomophagy.

One of the most important studies on sensitization to edible insects among the European population was conducted by Scala et al. [22]. The study involved 2014 patients. The patients who were the subjects of the study presented with a range of allergic conditions, including respiratory disorders, food allergies, and atopic dermatitis. All participants indicated that they had no prior experience with consuming edible insects. The aim of the study was to determine the prevalence of sensitization in three insect species: the house cricket (*Acheta domesticus*), the migratory locust (*Locusta migratoria*), and the mealworm (*Tenebrio molitor*).

**Figure 2:**
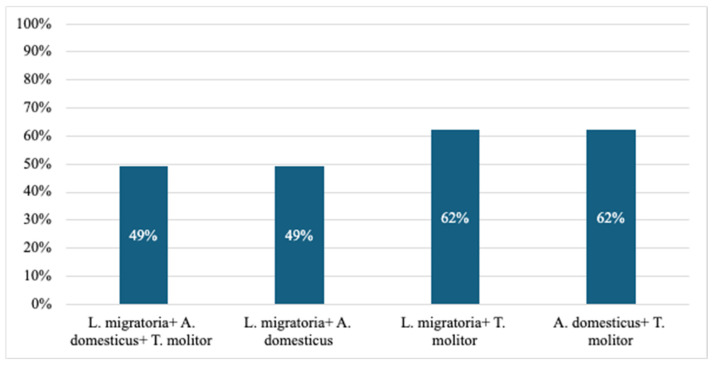
The percentage of people allergic to different combinations of two or three edible insects: *Locusta migratoria*, *Acheta domesticus* and *Tenebrio molitor*, as reported by Scala et al. [22].

Of the individuals tested, 195 (9.7%) demonstrated sensitization to at least one insect species. The house cricket was the most prevalent allergen, causing sensitization in 83% of those sensitized (161/195), followed closely by the mealworm, which caused reactions in 79% of those sensitized (154/195). The migratory locust allergen showed the lowest percentage of induced sensitization (51%, (100/195)) compared to the other edible insect allergens tested. The percentage distribution of people sensitized to two or three allergens is shown in Figure 2. Monosensitization, defined as sensitization to a single species of insect, was observed in 16.9% of individuals (33/195) who exhibited insect hypersensitivity.

Furthermore, the study sought to ascertain the allergens that were most frequently responsible for inducing sensitization in the participants. The two most significant allergens were tropomyosin and arginine kinase (AK), which were responsible for the sensitization of 34% and 18.5% of participants, respectively (these allergens will be further characterized below). Other proteins, including myosin light chain, troponin C, paramyosin, and sarcoplasmic calcium-binding protein, were responsible for less than 5% of the reactions.

A limitation of this study was that it was conducted on a group of people suffering from allergic diseases. It is recommended that a similar study be conducted on a population of healthy participants to obtain more generalizable conclusions.

## 7. Major Allergens in Case of Food Allergy to Edible Insects

A major allergen in the context of food allergy is one that induces an IgE-dependent reaction in more than 50% of sensitized individuals after they have eaten a particular food [44]. Based on this definition and the lack of sufficient research, it is not possible to clearly identify a major allergen in the context of allergies to edible insects. Nevertheless, tropomyosin appears to have the greatest association with allergic reactions, suggesting its potentially key role in causing allergies [7,20,22,27,29]. This allergen is responsible for approximately 34% of all cases of sensitization [22].

### 7.1. Tropomyosin

Tropomyosin is the major allergen among invertebrates. Tropomyosin is found in arthropod clusters, including crustaceans (shrimp, crabs, lobsters), insects (e.g., *Acheta domesticus*, *Locusta migratoria*, *Tenebrio molitor*, and *Alphitobius diaperinus*) and arachnids (e.g., house dust mites). The prevalence of tropomyosin in various invertebrate species renders it an important panallergen [45]. This protein is predominantly expressed in muscle cells. Tropomyosin is composed of two parallel alpha-helices that form a coiled-coil structure, which allows it to interact with actin. Actin plays a key role in regulating muscle contraction, cell shape and movement. Tropomyosin is characterized by a high degree of homology and a conservative structure, which results in frequent cross-reactions between different species or even clusters of invertebrates consumed. However, it does not show cross-reactivity with vertebrate tropomyosin [45].

### 7.2. Arginine Kinase (AK)

The second most significant allergen appears to be arginine kinase, which is responsible for approximately 18.5% of sensitization cases [22]. AK is an enzyme belonging to the phosphotransferase family, which plays a pivotal role in cellular energy metabolism, particularly in cells that require substantial energy (e.g., myocytes and neurons). It catalyzes the formation of phosphoarginine, an energy reservoir, by transferring a phosphate group from ATP to arginine [46].

This enzyme is widely distributed among arthropods (crustaceans, insects, arachnids), which can lead to cross-allergic reactions. Additionally, it can cause inhalant allergies [7]. It has been demonstrated that heat processing, including boiling, frying, and baking, may result in partial degradation of these allergens. However, this does not necessarily lead to the complete elimination of their IgE-binding capacity, which may contribute to the preservation of allergenicity [7].

### 7.3. Larval Cuticle Protein (LCP)

In diagnosing primary mealworm allergy, larval cuticle protein (LCP) should be mentioned. Immunoblotting studies confirmed IgE binding to LCP A1A in three out of four subjects, which indicates its potential significance as an allergen of mealworm disease. Therefore, it seems to be a specific protein in allergy to this insect and is considered a potential marker of primary allergy to this insect, although further studies on a larger group of people are necessary to confirm its role. The family of proteins to which LCP belongs is unknown; three isoforms of this protein are distinguished as follows: A1A, A2B and A3A. The route of exposure to larval cuticle protein includes both the oral and inhalation routes. LCP contains a conserved 64-amino-acid domain that binds chitin (R&R consensus), found in arthropods, which may indicate its important biological function. LCP may be the dominant allergen in primary mealworm allergy, which contrasts with cross-allergy in which other panallergens such as tropomyosin, arginine kinase, and myosin heavy chain predominate. Notably, LCP has not previously been identified as an allergen in insects or crustaceans, highlighting the need for further research in this area [47,48].

Currently, there is no possibility of testing larval cuticle protein or homologous proteins using IgE in available tests. The ability to detect this protein in the future may potentially bring new insights into allergies related to the mill moth, which could possibly contribute to a better understanding of allergic reactions associated with these proteins.

## 8. Cross-Reactivity of Edible Insects’ Allergens

As was highlighted above, the IgE-mediated sensitization and clinical manifestations of allergy to edible insects may be due either to primary sensitization or to cross-reactivity between allergens. The latter is the result of the widely spread presence of allergens that are common to multiple invertebrate species. Hence, severe symptoms, including anaphylaxis, following insect ingestion may occur in patients with no prior history of allergy to edible insects and in those who have not previously consumed edible insects, as evidenced by the findings from the available case reports and summarized by de Gier and Verhoeckx [1]. The collected data revealed an absence of allergic history in 54.3% (25 of 46) of subjects who had experienced anaphylaxis associated with the ingestion of edible insects. Another feature associated with allergic reactions to edible insects—and common with allergies to other foods—is the fact that they may occur after regular or occasional, repeated and uneventful exposure to a given allergen source during several years preceding the generalized reaction incident, as was described in the previously cited large study by Scala et al. [22] and in case reports [14,49,50,51]. Further support for the importance of potential cross-reactivity of insect allergens and allergens from other sources as an elicitor of local and systemic food allergy symptoms comes from a recent study in Poland, where analysis of more than 6000 reports from a multiplex IgE assay (ALEX2) revealed IgE sensitization to *Tenebrio molitor* mealworm in 4.3% of subjects with frequent co-sensitization to allergens coming from other invertebrates, such as the migratory locust (*Locusta migratoria*), crab (*Chionoecetes* spp.), house cricket (*Acheta domesticus*), fire ant (*Solenopsis* spp.), American cockroach (*Periplaneta americana*), common mussel (*Mytilus edulis*), northern shrimp (*Pandalus borealis*), oyster (*Ostrea edulis*), squid (*Loligo* spp.) and lobster (*Homarus gammarus*) [52]. Importantly, the assays reported in that study had been performed before the edible insects were widely present in nutritional products in Poland.

The main proteins originating from edible insects that are considered important in the context of eliciting food-dependent reactions include tropomyosin and arginine kinase; however, other allergens are considered potential culprits, including troponin C, myosin light and heavy chains, alpha-amylase, sarcoplasmic calcium-binding protein and others [1,47,52,53,54].

From the clinical point of view, the crucial issue is the possible cross-reaction of proteins ingested during consumption of edible insects with allergen components present in common sensitizers. In the following section, the most common potential cross-reactivities of edible insects’ proteins and commonly encountered allergens will be presented, with focus on their clinical aspects

### 8.1. Tropomyosin

The cross-reactivity between Der p 10, a tropomyosin of *Dermatophagoides pteronyssinus* house dust mite, and other known allergenic tropomyosins from, e.g., shrimps and cockroaches, has an acknowledged and established significance and its implications for allergy practice and the management of mite-sensitized subjects are well known [48]. With the growing appearance of edible insects-derived products in a global market, the question of cross-reactivity of their allergens is being raised [55]. Mealworms belong to the Arthropoda phylum, which implies the possibility of cross-reaction of their proteins with those from other arthropods [56]. This possibility is increased by the fact that several pan-allergens have previously been described within the *Arthropoda*, including tropomyosin, arginine kinase and glutathione S-transferase [45,57,58]. A study by Verhoeckx et al. [56] has confirmed that sera from patients who are allergic to house dust mites or crustaceans may cross-react with mealworm proteins, of which arginine kinase and tropomyosin have been identified as major cross-reactive allergens. Following these findings, Broekman et al. aimed to assess the allergenic potential of the *Tenebrio molitor* mealworm in the shrimp-allergic population [54]. In a study involving 15 patients who were allergic to shrimp and house dust mites, positive SPT and BAT in response to mealworm exposure were seen in all participants. DBPCFC confirmed the mealworm allergy in 13 patients; however, the remaining 2 patients did not complete the challenge due to severe reactions during the first stage, apparently resulting from consumption of the verum. Moreover, in a different study which included previously assessed mealworm-allergic patients [59], sensitization to other insects (house cricket, giant mealworm, lesser mealworm, African grasshopper, large wax moth and black soldier fly) was ascertained in all of them.

An investigation of a broadened population, including four groups with different clinical features (shrimp-allergic; HDM- but not Der p 10-allergic; seasonal rhinitis without sensitization to HDM or shrimp; non-atopic controls), was performed by the same group [60]. Among those allergic to HDM but not cross-reactive to shrimps, 22% had a positive sIgE test result in response to mealworm exposure. In the non-mite-allergic subjects with seasonal allergic rhinitis, only three (15%) tested positive for mealworm sIgE. These results suggest that allergens other than crustacean tropomyosin may be involved in cross-reactivity or co-sensitization between edible insects and other allergen sources, although to a lesser extent. This renders the spectrum of clinical co-sensitization possibilities far more complex and further underlines the necessity of thorough exploration of IgE-dependent cross-reactivity between insects and food and airborne allergens.

The results of the assessments of the allergenic potential of the *Acheta domesticus* [61] and *Gryllus bimaculatus* crickets [62], as well as of the black soldier fly (*Hermetia illucens*), [63] also suggest the considerable possibility of cross-reactivity between insects’ and shrimps’ tropomyosins. House cricket tropomyosin, contrary to tropomyosin of the *Litopenaeus vanamei* shrimp, shows high stability with regard to gastrointestinal digestion, which renders it a possible culprit with regard to both primary and cross-sensitization as a sequel to edible cricket consumption [61].

### 8.2. Arginine Kinase (AK)

AK has been identified through multiple studies as another major allergen of various species of edible insects (reviewed by Ribeiro et al. [43]). Its potential clinical relevance was established using both laboratory (immunoblot, immunoprecipitation, liquid chromatography, BAT) and/or clinical (SPT, DBPCFC) procedures with regard to yellow mealworm *(Tenebrio molitor)* [54,56,64], field cricket (*Gryllus bimaculatus*) [65], Bombay locust (*Patanga succinta*) [66] and silkworm (*Bombyx mori*) [67,68]. The *Bombyx mori* silkworm, although not approved for human consumption in the EU, is being evaluated as a potentially applicable source of nutrients in humans [69]. Despite the lack of approval for alimentary use in humans in Europe, sensitization may still occur after accidental or deliberate consumption of silkworm, which is widely popular as a snack or street food in popular tourist destinations in southeast Asia. Considering this fact, in addition to the above-highlighted cross-reactivity, the possibility of sensitization and symptomatic allergy to edible insects other than EU-approved ones cannot be neglected or omitted during the diagnostic process.

Notably, anaphylaxis does not only occur in tandem with sensitization to major shellfish allergens (tropomyosin, arginine kinase, etc.). Sensitization to minor allergens (e.g., sarcoplasmic calcium-binding protein and myosin chains) although reported to be less frequent [70,71,72,73,74], can also be a causative factor of generalized allergic reaction [75,76]. Considering potential cross-reactivities, the need for awareness-raising actions with regard to the possible presence of shellfish and dust mite allergens in products derived from edible insects, is further justified.

## 9. Diagnosis of Allergy to Edible Insects

Since the appearance of symptoms attributable to the ingestion of edible insects may result from both primary and cross-reactive sensitization, both aspects need to be taken into consideration while assessing patients who present such symptoms. In hitherto published case reports, allergy and sensitization to edible insect allergens have been assessed with the use of SPT, BAT, immunoblot, ELISA, as well as inhalational and oral provocation tests (as reviewed by de Gier & Verhoeckx [1]) Commercially available tests for serum-specific IgE assessment have been designed based on the use of whole insect extracts and not specific allergenic proteins. However, for the purpose of detecting IgE-mediated sensitization to selected EU-approved edible insects, the SPT and serum specific IgE tests have been specifically prepared by the respective companies.

Currently, diagnostic opportunities in case of suspected allergy to edible insects are somewhat limited. Specific IgE in serum can be measured using the ImmunoCAP tests authorized for use in diagnostics (Phadia AB, Uppsala, Sweden). With regard to edible insects approved as human food in the EU, only kits for mealworm (*Tenebrio mollitor*) sIgE are available for diagnostic use. Regarding other insects potentially used in the food industry, or approved for human consumption in other countries, silkworm (*Bombyx mori*) sIgE can be measured through ImmunoCAP as well [77].

The ImmunoCAP ISAC assay is a semi-quantitative immunoassay that allows for simultaneous measurement of specific IgE antibodies in association with 112 different allergen components. Currently, it does not include components from edible insects approved for direct consumption in the EU or potentially used in the food industry [77].

ALEX^2^ (Macro Array Diagnostics GmbH, Vienna, Austria) is a modified version of ALEX (Allergy Xplorer)—an ELISA-based in vitro multiplex allergy test allowing (in the current version) simultaneous measurements of sIgEs against 295 allergen extracts and allergen components. In EU-approved edible insects, sIgE measurement in house cricket (*Acheta domesticus*), mealworm (*Tenebrio mollitor*) and migratory locust (*Locusta migratoria*) allergen extracts can be assessed with the use of ALEX^2^. IgEs specific to *Bombyx mori* or other edible insects cannot be measured; nor can be any edible insects’ allergen components [78].

## 10. Diagnosis of Edible Insect Allergens’ Cross-Reactivity

As mentioned above, the edible insect allergens that may be considered the culprits in IgE-mediated reactions belong to tropomyosins and arginine kinases. Additionally, alpha-amylase has been identified as a novel allergenic protein in patients exhibiting signs of respiratory allergy to *Tenebrio molitor* mealworm in an occupational setting [53]. Extensive possibilities of cross-reactions may render it difficult to accurately identify an allergen directly responsible for symptoms’ elicitation. However, attributing the symptoms’ appearance to edible insect allergens may be facilitated if there is conclusive historical data and clearly ascertainable IgE-mediated sensitization to known cross-reactive allergens. The most appealing theoretical exemplification of such a possibility is the presence of IgE specific to tropomyosin from various sources in association with confirmed exposure to edible insects leading to the development of allergy symptoms.

In daily practice within a clinical setting, preparation of skin test solutions or sIgE detection kits designed for use in a single patient is obviously not possible. Therefore, accessibility of the assessment of IgEs specific for tropomyosin and other allergenic components cannot be overestimated in such a context. In addition to IgE binding to tropomyosin and arginine kinase, IgE binding to many different proteins from the mealworm has been described. These proteins include paramyosin, chitinase, troponin C, myosin light and heavy chain, hexamerin, α-amylase, trypsin-like proteinase, cockroach allergen, and larval cuticle protein [48]. Among these other molecules, the ALEX2 test allows for the detection of homologous molecules such as paramyosin (heavy myosin chain—Der p 11—*Dermatophagoides. pteronyssinus*), troponin C (Cra c 6—*Crangon crangon* [shrimp]), and myosin (Pen m 3—*Penaeus monodon* [shrimp]). Proteins such as paramyosin and chitinase, which are found in both edible insects and other allergens, share a sequence identity ranging from 35% to 90%. However, the clinical relevance of cross-reactivity between these proteins and their homologs in edible insects has not yet been conclusively demonstrated [48].

With the use of ImmunoCAP, it is possible to assess the presence of sIgEs with regard to single allergenic tropomyosins derived from *Dermatophagoides pteronyssinus* house dust mites (Der p 10) and *Penaeus aztecus* shrimp (Pen a 1). In addition, assessment of sIgE obtained from extracts of the German cockroach (*Blatella germanica*) and American cockroach (*Periplaneta americana)* may be helpful for diagnosing possible tropomyosin cross-reactivity.

Implementation of the ImmunoCAP ISAC assay allows for the assessment of IgEs specific to tropomyosins of *D. pteronyssinus* mite (Der p 10), *Penaeus monodon* shrimp (Pen m 1), *Blatella germanica* German cockroach (Bla g 7) and *Anisakis simplex*—a fish parasite that belongs to Nematoda phylum (Ani s 3).

The ALEX^2^ multiplex assay includes tropomyosins potentially cross-reacting with edible insect allergens derived from the following sources: *D. pteronyssinus* mite (Der p 10), *Blomia tropicalis* mite (Blo t 10), American cockroach (Per a 7), *Anisakis simplex* (Ani s 3) and *P. monodon* shrimp (Pen m 2).

ImmunoCAP ISAC also allows for assessment of sIgE to arginine kinase (the other allergenic proteins potentially responsible for eliciting cross-reactions after the consumption of edible insects) originating from *P. monodon* shrimp (Pen m 2). Arginine kinases included in the ALEX^2^ panel of allergen components include the ones originating from *D. pteronyssinus* mite (Der p 20), the German cockroach (Bla g 9) and *P. monodon* shrimp (Pen m 2).

## 11. Summary and Conclusions

Edible insects are distinguished by a high concentration of protein, essential fatty acids, vitamins, and micronutrients. The farming of these insects requires a significantly smaller input of resources, including water, breeding space, and feed, in comparison to traditional livestock farming. As projected by the FAO, the global population is expected to reach 9 billion by 2050. It can be reasonably deduced that entomophagy may prove to be a significant factor in addressing the impending global food crisis. Nevertheless, despite the optimistic prognosis, the potential risks associated with insect consumption, including the occurrence of allergic reactions, must be considered. The primary insect allergens are tropomyosin and arginine kinase. Given the close phylogenetic affinity between insects and arthropods, there is a risk of cross-reactions between edible insect allergens and the allergens originating from crustaceans and house dust mites. The introduction of edible insects in the European Union in 2021 is a relatively recent phenomenon, which has resulted in a paucity of research on the safety of their consumption, particularly in the context of food allergies. One of the most significant research challenges is the absence of a comprehensive nomenclature of edible insect allergens within the IUIS (International Union of Immunological Societies) database. Furthermore, additional research is necessary to elucidate the impact of industrial heat treatment methods, such as cooking and frying, on the allergenicity of insect proteins and the influence of their diet on their allergenic properties. Ultimately, edible insects have the potential to contribute significantly to a sustainable food production system. Nevertheless, a comprehensive understanding of their safety for consumers, particularly in the context of allergies, necessitates further investigation.

## Figures and Tables

**Table 1:** The primary allergens of Acheta domesticus, their function, and the impact of heat treatment on their allergenic potential.

References	Protein	Functions of the Protein in Insect	Effects of Thermal Processing
[7]	Tropomyosin	Muscle protein	Extremely resistant to thermal processing. Tropomyosin remains immunoreactive after heat treatments such as boiling and baking.
[7]	Arginine kinase	Energy metabolism enzyme	Intense processing methods, such as prolonged boiling and high-temperature frying, may reduce its allergenicity.
[7]	Hexamerin 1B	Transport and storage protein	Intense processing methods, such as prolonged boiling and high-temperature frying, may reduce its allergenicity.
[7]	Glyceraldehyde 3-phosphate dehydrogenase	Enzyme involved in glycolysis	Intense processing methods, such as prolonged boiling and high-temperature frying, may reduce its allergenicity.
[7]	Chitinase	Chitin-degrading enzyme	Intense processing methods, such as prolonged boiling and high-temperature frying, may reduce its allergenicity.
[7,22]	Paramyosin	Muscle protein	Intense processing methods, such as prolonged boiling and high-temperature frying, may reduce its allergenicity.
[20]	Hemocyanin	Respiratory protein	Relatively resistant to thermal processing. It may retain partial allergenicity after heat treatment.
[20]	Vitellogenin	Yolk protein	Intense processing methods, such as prolonged boiling and high-temperature frying, may reduce its allergenicity.
[20]	Troponin I	Muscle protein	Intense processing methods, such as prolonged boiling and high-temperature frying, may reduce its allergenicity.
[20,22]	Myosin light chain	Muscle protein	Intense processing methods, such as prolonged boiling and high-temperature frying, may reduce its allergenicity.
[20]	Apolipophorin-III	Transport protein	Intense processing methods, such as prolonged boiling and high-temperature frying, may reduce its allergenicity.
[22]	Troponin C	Calcium-binding protein	Intense processing methods, such as prolonged boiling and high-temperature frying, may reduce its allergenicity.
[22]	Sarcoplasmic calcium-binding protein	Calcium-binding protein	Intense processing methods, such as prolonged boiling and high-temperature frying, may reduce its allergenicity.

**Table 2:** Primary allergens of *Tenebrio molitor*, their function, and the impact of heat treatment on their allergenic potential.

References	Protein	Functions of Protein in Insect	Effects of Thermal Processing
[23]	Tropomyosin	Muscle protein	Extremely resistant to thermal processing. Tropomyosin remains immunoreactive after heat treatments such as boiling and baking.
[23]	Arginine kinase	Energy metabolism enzyme	Intense processing methods, such as prolonged boiling and high-temperature frying, may reduce its allergenicity.
[23]	Heat shock protein 70	Heat shock protein	Relatively resistant to thermal processing. It may retain partial allergenicity after heat treatment.
[23]	α-Amylase	Enzyme that breaks down starches	Intense processing methods, such as prolonged boiling and high-temperature frying, may reduce its allergenicity.
[23]	Apolipophorin-III	Transport protein	Intense processing methods, such as prolonged boiling and high-temperature frying, may reduce its allergenicity.
[23]	Hemolymph protein 12 kDa	Storage protein	Intense processing methods, such as prolonged boiling and high-temperature frying, may reduce its allergenicity.
[23]	Protein of the larval cuticle	Structural protein of the cuticle	Relatively resistant to thermal processing. It may retain partial allergenicity after heat treatment.
[24]	Hexamerin 1B	Transport and storage protein	Intense processing methods, such as prolonged boiling and high-temperature frying, may reduce its allergenicity.
[25]	Chitinase	Chitin-degrading enzyme	Intense processing methods, such as prolonged boiling and high-temperature frying, may reduce its allergenicity.
[25]	Glutathione S-transferase	Detoxification enzyme	Intense processing methods, such as prolonged boiling and high-temperature frying, may reduce its allergenicity.
[22]	Troponin C	Calcium-binding protein	Intense processing methods, such as prolonged boiling and high-temperature frying, may reduce its allergenicity.
[22]	Paramyosin	Muscle protein	Intense processing methods, such as prolonged boiling and high-temperature frying, may reduce its allergenicity.
[22]	Myosin light chain	Muscle protein	Intense processing methods, such as prolonged boiling and high-temperature frying, may reduce its allergenicity.
[22]	Sarcoplasmic calcium-binding protein	Calcium-binding protein	Intense processing methods, such as prolonged boiling and high-temperature frying, may reduce its allergenicity.

**Table 3:** Primary allergens of *Locusta migratoria*, their function, and the impact of heat treatment on their allergenic potential.

References	Protein	Functions of Protein in Insect	Effects of Thermal Processing
[27]	Tropomyosin	Muscle protein	Extremely resistant to thermal processing. Tropomyosin remains immunoreactive after heat treatments such as boiling and baking.
[27]	Arginine kinase	Energy metabolism enzyme	Intense processing methods, such as prolonged boiling and high-temperature frying, may reduce its allergenicity.
[27]	S-glutathione transferase	Detoxification enzyme	Intense processing methods, such as prolonged boiling and high-temperature frying, may reduce its allergenicity.
[27]	Chitinase	Chitin-degrading enzyme	Intense processing methods, such as prolonged boiling and high-temperature frying, may reduce its allergenicity.
[27]	Cross-reactive carbohydrate determinants	Structural elements of glycoproteins	Intense processing methods, such as prolonged boiling and high-temperature frying, may reduce its allergenicity.
[17]	Hemocyanin	Respiratory protein	Relatively resistant to thermal processing. It may retain partial allergenicity after heat treatment.
[28]	Heat shock protein 70	Heat shock protein	Relatively resistant to thermal processing. It may retain partial allergenicity after heat treatment.
[28]	Hexamerin	Transport and storage protein	Intense processing methods, such as prolonged boiling and high-temperature frying, may reduce its allergenicity.
[28]	Serine protease	Protein degrading enzyme	Intense processing methods, such as prolonged boiling and high-temperature frying, may reduce its allergenicity.
[28]	Trypsin	Protein degrading enzyme	Intense processing methods, such as prolonged boiling and high-temperature frying, may reduce its allergenicity.
[22]	Troponin C	Calcium-binding protein	Intense processing methods, such as prolonged boiling and high-temperature frying, may reduce its allergenicity.
[22]	Paramyosin	Muscle protein	Intense processing methods, such as prolonged boiling and high-temperature frying, may reduce its allergenicity.
[22]	Myosin light chain	Muscle protein	Intense processing methods, such as prolonged boiling and high-temperature frying, may reduce its allergenicity.
[22]	Sarcoplasmic calcium-binding protein	Calcium-binding protein	Intense processing methods, such as prolonged boiling and high-temperature frying, may reduce its allergenicity.

**Table 4:** Primary allergens of *Alphitobius diaperinus*, their function, and the impact of heat treatment on their allergenic potential.

References	Protein	Functions of Protein in Insect	Effects of Thermal Processing
[25,29]	Tropomyosin	Muscle protein	Extremely resistant to thermal processing. Tropomyosin remains immunoreactive after heat treatments such as boiling and baking.
[25,29]	Arginine kinase	Energy metabolism enzyme	Intense processing methods, such as prolonged boiling and high-temperature frying, may reduce its allergenicity.
[25]	Chitinase	Chitin-degrading enzyme	Intense processing methods, such as prolonged boiling and high-temperature frying, may reduce its allergenicity.
[25]	Glutathione S-transferase	Detoxification enzyme	Intense processing methods, such as prolonged boiling and high-temperature frying, may reduce its allergenicity.

## Data Availability

No new data were created or analyzed in this study. Data sharing is not applicable to this article.
